# Recruiting to inpatient-based rehabilitation trials: lessons learned

**DOI:** 10.1186/s13063-015-0588-2

**Published:** 2015-03-04

**Authors:** Sarah F Tyson, Nessa Thomas, Andy Vail, Pippa Tyrrell

**Affiliations:** School of Nursing Midwifery & Social Work, University of Manchester, Manchester, UK; Stroke Research Centre, University of Manchester, Manchester, UK; Centre for Biostatistics, University of Manchester, Manchester, UK; Salford Royal Foundation NHS Trust, Stott Lane, Salford, M6 8D UK; Manchester Academics Health Sciences Centre, Manchester, UK; Jean McFarlane Building University of Manchester, Oxford Rd, Manchester, M13 9PL UK

**Keywords:** Stroke, Rehabilitation, Trial, Recruitment

## Abstract

**Abstract:**

Effective recruitment is central to successful trials but is often problematic. This article reports the lessons learnt while recruiting stroke rehabilitation patients to a multi-centre randomised control trial. As intended, 94 participants were recruited from 12 inpatient stroke rehabilitation services in Northwest England over 12 months; however, recruitment rates were highly varied (from 0.6 to 2.5 participants per site per month) as were the nature of the stroke services and the personnel available. Consequently, bespoke recruitment procedures were needed at each site. As the assessment skills needed to screen for the selection criteria were specific to therapists, our most common strategy was for the hospital therapists to screen patients and make referrals directly to the trial team. However, we identified several strategies undertaken by the research nurse in the highest recruiting site that appeared to positively impact on recruitment. These strategies included involving the whole multidisciplinary team, being part of the stroke team, facilitating contact between the clinical and trial teams and using inclusive recruitment and watchful waiting strategies.

Rehabilitation trials frequently require skilled assessments by therapists, rather than by doctors or nurses to identify potential participants. Thus, research support models need to include suitably skilled trial therapists. Recruitment can be enhanced by enthusiastic, regular and structured engagement with the entire stroke multidisciplinary team and by using inclusive recruitment and ‘watchful waiting’ strategies to identify and monitor potential participants.

**Trial registration:**

ISRCTN29533052. Registered May 2011

## Background

Randomised controlled trials (RCTs) are the standard criterion for assessing healthcare interventions, but effective recruitment is required to maintain statistical power, ensure external validity and prevent unnecessary costs and delays. This is a challenge that few trials overcome [1]. There is a growing body of literature highlighting these difficulties and suggesting solutions in different settings and clinical groups, although strategies for best practice are yet to be established [2-5].

As stroke rehabilitation trialists, we need to know how best to recruit eligible participants, whether from inpatient stroke units or following discharge to community settings. Inclusion criteria for rehabilitation trials tend to include objective measures of activity limitations (for example, walking speed) or enduring impairments (for example, arm weakness), as well as clinical diagnosis. Such information is rarely recorded in medical records, which mean conventional recruitment techniques, such as screening case notes are ineffective.

Here we report the lessons learnt from a stroke-rehabilitation RCT on effective recruitment strategies. We aimed to recruit 90 patients over a 12-month period for randomisation to a trial where they would receive 4 weeks of patient-led upper limb mirror therapy or lower limb exercises (the attentional control) in 12 inpatient stroke-rehabilitation units in Northwest England. Ethical approval was provided by Lancaster National Research Ethics Committee (Registration number 10/H1015/85). All participants gave informed consent.

## Main text

We based our expected recruitment rate (one participant per site per month) on service activity records, our experience of previous studies in the participating hospitals, and published data regarding the frequency of impairments and activity limitations after stroke. Stroke Research Network staff identified potential participants from stroke unit admission data, screened against eligibility criteria (with the help of stroke therapists, if necessary), and referred eligible patients to the trial team, whose members would seek consent and then perform baseline assessments before randomisation.

Although all sites were able to recruit, recruitment rates differed markedly, ranging from 0.6 to 2.5 participants per site per month (Table 1), making it difficult to predict recruitment potential for this, or future trials. The size, nature and organisation of the stroke services and staff skill mix were also highly varied, as were the personalities involved and the team climate within the service. There was no obvious organisational or clinical explanation for the variability in recruitment rate, and in effect, a bespoke strategy needed to be developed at each site.

**Table 1:** **Details of recruitment at each participating site**

**Site**	**Participants/month**	**Total recruited**	**Month 1**	**Month 2**	**Month 3**	**Month 4**	**Month 5**	**Month 6**	**Month 7**	**Month 8**	**Month 9**	**Month 10**	**Month 11**
Site 1	0.8	9	4	0	0	0	2	1	1	0	1	0	0
Site 2	0.9	10	1	0	0	0	1	1	3	0	2	1	1
Site 3	0.7	8	1	1	1	1	0	0	2	0	0	2	0
Site 4	2.5	25		4	3	5	4	3	1	0	2	2	1
Site 5	0.5	5		2	1	0	1	0	1	0	0	0	0
Site 6	0.8	8		3	1	1	2	0	0	1	0	0	0
Site 7	0.4	4			1	0	1	0	1	0	0	1	0
Site 8	1.1	9				2	1	2	1	1	1	1	0
Site 9	0.6	5				1	0	1	1	0	2	0	0
Site 10	0.4	3					1	0	1	0	1	0	0
Site 11	0.5	3						1	1	0	1	0	0
Site 12	2.5	5										0	5
Total			6	10	7	10	13	9	13	2	10	7	7

The clinical teams’ research experience, skills and enthusiasm, and the research network staff’s clinical and assessment skills impacted strongly on the tasks that individuals could be asked to perform. In most cases, the research network staff did not have the assessment skills to screen for potential participants, as these assessments are usually the domain of stroke therapists. Consequently, our most common strategy was for the hospital therapists to screen patients and then liaise directly with the trial team. Research network staff’s confidence to extend their sphere of influence by engaging with the stroke therapists varied. Again, in some instances, the solution was for the trial team to work directly and build a relationship with the clinicians.

One site (Site 4, Table 1) had a notably higher recruitment rate than the other sites throughout the trial, recruiting 25% of all participants. During informal discussions with the clinicians and research nurses at this site (and others), we identified several differences in approach used by the research network nurse in Site 4, which appeared to positively impact the recruitment rate. These are detailed below.

### Clinician-related strategies

The research nurse at Site 4 ensured she had a good knowledge of the whole stroke service (rather than focussing on admissions), built an effective working relationship with all members of the multidisciplinary stroke team (rather than just the principal investigator and research staff) and was perceived as part of the team by being seen on the ward daily and assisting when appropriate. This made involvement in research and recruitment a role for all team members She gave frequent briefings; held regular, structured liaison meetings with the team regarding recruitment rates and progress of the trial; and kept a ‘research board’ on the ward up-to-date with progress. She also facilitated on-going contact between the clinical and trial teams. This helped the clinical team maintain their attention on recruitment and better understand the trial and their role, which created a sense of allegiance to the trial.

### Patient-related strategies

The research nurse at Site 4 took a more inclusive approach than most. It was her view that, wherever possible, participation should be the patients’, rather than the healthcare professionals’ choice. She viewed all patients as potential participants unless/until there was an overwhelming reason for why they should not be considered, rather than excluding on potential reasons. If in doubt, she discussed it with the principal investigator, trial team or offered the trial to the patient so they could make the choice. The trial’s service user consultation group was fully supportive of this approach, expressing dismay at the notion of ‘gate-keeping’ and ‘cherry-picking’.

Like many rehabilitation trials, the nature of our selection criteria meant patients who were unsuitable to participate early after their stroke may become eligible at a later stage. A ‘watchful waiting’ strategy (that is, monitoring patients’ progress and approaching them if/when they improved or their condition changed) required meticulous record keeping and ongoing discussions within the multidisciplinary team, but enabled many more suitable potential participants to be identified in the post-acute stage of stroke.

At the other recruitment sites, these strategies were adopted to some degree. Site 12 (Table 1) was the last site to start recruiting, due to long delays obtaining approvals, and adopted the strategies described above with similar success, albeit only for a short period.

To the authors’ knowledge, this is the first paper to specifically explore recruitment to inpatient rehabilitation trials involving a multidisciplinary stroke team; the findings and solutions may be generalisable to rehabilitation trials in other clinical groups. We found that recruitment to rehabilitation trials requires assessment skills that are beyond the scope of most nurses and many doctors, and information is rarely available in clinical notes. Thus, research support needs to ensure a workforce with a suitable skill mix. Like rehabilitation trialists in other settings [6], we felt this support could be most effectively and efficiently provided by dedicated research therapists working across the research sites rather than generic staff working on multiple trials at a single site.

Perhaps our most striking finding is that a few apparently simple, no-cost strategies appeared to positively affect recruitment rates. An inclusive recruitment strategy, ‘watchful waiting’, and a close relationship between the research nurse and the clinical team achieved a recruitment rate in Site 4 that was twice as high as the average for the trial. If this observation could be generalised, there is potential to dramatically reduce the cost and duration of clinical trials and the time scale that will impact clinical practice. However, we fully acknowledge the limitations of the narrative nature of this report, which merely indicates that further research is warranted.

There are clear parallels between the challenges of rehabilitation trials and other complex interventions, such as surgery and oncology, where research has tended to focus on patient- and design-related factors [6-9]. Recently, Donovan *et al*. [10] explored staff-related issues, describing how staff’s clinical and professional priorities, responsibilities and beliefs can inadvertently contribute to recruitment difficulties. Our experience supports these findings and adds to them by suggesting that simple changes to operational processes may enhance recruitment rates. Like Donovan *et al*. [10], we recommend support and training to enable staff to develop skills and confidence. In our experience, mere dissemination and discussion highlighting the potential benefits of these processes and how they can be achieved is largely ineffective.

## Conclusions

Recruitment to rehabilitation trials often requires therapists’ assessment skills rather than those provided by doctors or nurses, which, in turn, necessitate a flexible research support workforce, including dedicated trial therapists. Recruitment can be further enhanced by engaging the multidisciplinary stroke team, and using inclusive recruitment and ‘watchful waiting’ strategies to identify and monitor potential participants.
